# Characteristics of tRNA-Derived Small RNAs and microRNAs Associated with Immunocompromise in an Intrauterine Growth-Restricted Pig Model

**DOI:** 10.3390/ani12162102

**Published:** 2022-08-17

**Authors:** Jianfeng Ma, Mailin Gan, Jingyun Chen, Lei Chen, Ye Zhao, Yan Zhu, Lili Niu, Shunhua Zhang, Yanzhi Jiang, Zongyi Guo, Jinyong Wang, Li Zhu, Linyuan Shen

**Affiliations:** 1 Department of Animal Science, College of Animal Science and Technology, Sichuan Agricultural University, Chengdu 611130, China; 2 Farm Animal Genetic Resource Exploration and Innovation Key Laboratory of Sichuan Province, Sichuan Agricultural University, Chengdu 611130, China; 3 Chongqing Academy of Animal Science, Chongqing 402460, China; 4 College of Life Science, China West Normal University, Nanchong 637009, China; 5 Department of Zoology, College of Life Science, Sichuan Agricultural University, Ya’an 625014, China

**Keywords:** intrauterine growth restriction, pig model, spleen, immunocompromise, microRNA, tsRNA

## Abstract

**Simple Summary:**

Intrauterine growth restriction (IUGR) refers to the slow growth and development of an embryo or fetus in the uterus of mammals. IUGR newborns commonly present with slow growth and the development of the body and organs accompany increased risks of infection during the early life period. IUGR remains a significant global public health issue, particularly in developing countries. In this work, we investigated the transfer RNA-derived small RNA and microRNA expression profiles in the spleen using pigs as an IUGR model. These results uncover an important potential regulator network involved in immunocompromise caused by IUGR. The present studies provide a novel perspective on the molecular regulatory mechanism of IUGR and a reference for prevention and treatment.

**Abstract:**

Intrauterine growth restriction (IUGR) is an important cause of newborn morbidity and mortality in mammals. Transfer RNA-derived small RNA (tsRNA) has become an emerging non-coding RNA in recent years. tsRNA and microRNAs (miRNAs) share similar mechanisms, which are involved in various biological processes. In this study, the pig was used as a model of IUGR, and the tsRNA and miRNA expression profile in the spleen was characterized by RNA sequencing. A total of 361 miRNAs and 620 tsRNAs were identified, of which 22 were differentially expressed miRNA (DEM) and 25 differentially expressed tsRNA (DET). tRF-5c were the primary tsRNA type making up more than 90%, and the most abundantly expressed tsRNAs are from tRNA-Gly-GCC. Functional enrichment analysis found that those DETs and DEMs have been implicated in the immune system process. Protein–protein interaction (PPI) network analysis revealed ssc-miR-370, ssc-miR-206, tiRNA-Ser-TGA-001 and tRF-Val-AAC-034 could be major regulators. TNF, TLR4, CD44, MAPK1 and STAT1 were predicted hub target genes. Those DETs and DEMs may regulate the T-cell receptor signaling pathway and Toll-like receptor signaling pathway to mediate the immunocompromise caused by IUGR. The results discussed in this article uncover the potential role of tsRNAs and miRNAs in IUGR porcine spleen.

## 1. Introduction

Intrauterine growth restriction (IUGR) refers to the slow growth and development of an embryo or fetus in the uterus of mammals. An individual whose birth weight is two standard deviations less than the population means or 10% less than the birth weight of normal newborns was defined as IUGR [1]. IUGR remains a public health concern worldwide, whose incidence can reach up to 15% in developing countries [2]. There are several reasons for IUGR, including fetal, placental and maternal [3]. IUGR is one of the most important causes of increased mortality and morbidity during peripartum [4]. IUGR newborns commonly present with slow growth and development of the body and organs, accompanied by increasing risks of infection during the early life period [5,6]. An ample number of studies have shown that human [7,8] and animal models [9,10,11] with IUGR are impaired in metabolic function, cardiovascular function, intestinal barrier and immune system.

Immunological dysfunctions are one of the characteristics of IUGR individuals and lead to complications. Studies have reported that thymic atrophy occurs in IUGR fetuses; the thymus is a primary immune organ to support T-cell development [12]. In animal models, lymphocytopenia in the thymus and lower cytokine levels in peripheral blood have been observed in sheep with IUGR [13]. In addition to the thymus, the spleen is also a main reservoir for lymphocytes [14]. The spleen is the largest body immune organ and contains both lymphocytes and macrophages, comprising a quarter of the lymphatic tissue [15]. Currently, there are not many researches about the IUGR, and the majority of studies on IUGR focus on certain tissues, such as the intestine [16,17], placenta [18], liver [19,20] and muscles [21], whereas studies referring to the spleen are relatively scarce. Spleen development is essential for establishing the acquired immunity of newborns. Hence, it is necessary to deeply investigate the changes in the spleen caused by IUGR. In recent years, researchers have explored the role of nutrients to improve the physical functioning of IUGR individuals [22,23]. Insights into the molecular mechanisms of IUGR may be helpful to develop novel targeted therapeutic molecules.

Epigenetic regulation has been demonstrated to participate in multiple biological processes [24]. Increasing research suggests that non-coding RNA plays an important role in epigenetic regulation [25]. microRNAs (miRNAs) are the most studied non-coding RNAs with 22 nucleotides in average length. miRNAs are involved in the regulation of gene expression at the post-transcriptional level, and have become a potential therapeutic and diagnostic target for various diseases [26]. The role of miRNAs in fetal growth and development and IUGR newborns has been reported in some studies. The abnormal expression of miR-29a in IUGR neonates has been reported to be associated with impaired intestinal barrier function [27]. Saget et al. reported miR-19a-3p was involved in insulin resistance in mice with IUGR [28]. However, to date, the miRNA-expressed profile in the spleen, related to IUGR, has yet to be reported. tRNA-derived small RNA (tsRNA) is a novel epigenetic regulator discovered in recent years, derived from precursor or mature transfer RNA (tRNA) [29]. tsRNA were assumed to be a ‘noise sequence’ in early studies [30,31]. With the development of high-throughput sequencing technology, the regulatory functions of tsRNA have been proven, including the regulation of mRNA stability [32], ribosome biogenesis [33] and translation initiation factor [34]. tsRNA has a similar function with miRNA and can regulate the gene expression at the post-transcription level [35], and they have recently attracted considerable attention from the research community. Studies has suggested that tRNAs are cleaved to generate tsRNA when cells are subjected to cellular stress [36]. tsRNA is regarded as a novel biomarker for multiple cancer and other diseases, whereas tsRNAs associated with IUGR have yet to be reported.

Animal models are necessary to study the occurrence and prevention of IUGR. The pig is not only an important agricultural animal, but also a good animal model of disease in biomedicine [37]. As a multi-fetal mammal, pigs have the highest incidence of IUGR among livestock species [19]. Pigs affected by IUGR have impaired health, slow growth and metabolic disorders [38]. These features closely mimic the IUGR of humans. Hence, pigs are considered to be a reliable and acceptable animal model for translational research into IUGR [39].

In this study, we use the pig as a model to investigate the expression characteristics of miRNA and tsRNA in the spleen associated with immunocompromise caused by IUGR. The findings from this article will provide a novel insight for the regulatory mechanisms of gene expression of IUGR and a theoretical basis in the prevention and treatment of IUGR.

## 2. Materials and Methods

### 2.1. Ethics Statement

Experiments involving animals carried out in strict accordance with the Regulations on the Administration of Laboratory Animals (Ministry of Science and Technology of China, revised in June 2004). All procedures in the present study were approved by the Animal Ethical and Welfare Committee of the Sichuan Agricultural University, Sichuan, China (approval number DKY-B20131403).

### 2.2. Animals and Sample Collection

A total of 12 half-sibs female DLY pigs were used in this study. Pigs were separated into two groups according to birth weight: Normal pigs (1.60 ± 0.05, *n* = 6) and IUGR pigs (1.07 ± 0.04, *n* = 6). The birth weight of normal pigs is close to the herd average (within 0.50 of a standard deviation). Piglet was defined as IUGR when birth weight less than the two standard deviations for the herd average. Piglets were weaned at the age of postnatal day 23. Piglets were weighed at weaning, at 3, 7, 14 days after weaning (daily at 8:00 a.m.), and peripheral blood was collected at weaning and 14 days after weaning. Pigs were slaughtered according to a standard commercial procedure and collected spleen on 14 days postweaning. The blood sample was collected in vacuum tubes with sodium heparin. After standing for 1 min, blood samples were centrifuged at 3000× *g* for 15 min. Blood plasma was collected and stored at −80 °C.

### 2.3. Peripheral Blood T-lymphocyte Subset Analysis and Cytokine Tests

Blood was transported to the laboratory within 2 h after collection, and immediately performed flow cytometry analysis (FACSCanto, Becton Dickinson, San Jose, CA, USA). A total of 100 ul of the blood was placed into 1.5 mL centrifuge tube, and then were stained with mouse anti-swine CD3-SPRD (catalogue #4510-13), CD4-FITC (catalogue #4515-02) and CD8-PE (catalogue #4520-09). The percentage of the T-lymphocyte subset were determined using CellQuest software (BD Biosciences, Franklin Lakes, NJ, USA). All antibodies were purchased from Southern Biotechnology Associates (Birmingham, AL, USA). The concentrations of C3 (Cat No. H186-1), IgG (Cat No. H106-1) and IgM (Cat No. H109-1) in the plasma were tested using the automatic biochemistry analyzer (HITACHI 3100, Tokyo, Japan). The test kits were purchased from Nanjing Jiancheng Bioengineering Institute (Nanjing, China).

### 2.4. miRNA and tsRNA Sequencing

We randomly selected 3 normal pig (birth weight 1.62 ± 0.02) and 3 IUGR pig (birth weight 1.08 ± 0.05) from the above-mentioned 12 pigs for small RNA sequencing. Total RNA in the spleen was extracted using TRIZOL reagent (Invitrogen, Guangzhou, China), according to the manufacturer’s instructions. Small RNA libraries were constructed according to previously reported procedures [40]. Briefly, total RNAs were denatured at 70 °C for 5 min and separated by a 15% TBE-urea gel with 10/60 oligo length standard ladder (Integrated DNA Technologies, Coralville, LA, USA). Small RNAs (15–40 nucleotides) were recovered by small RNA PAGE recovery kit (Zymo Research, Irvine, CA, USA). The small RNA was submitted for library preparation using NEB small RNA library kit (New England Biolabs, Ipswich, MA, USA). Sequencing was performed on an NextSeq 500 instrument following the manufacturer’s instructions (Illumina, San Diego, CA, USA). The analysis of small RNA-seq data refers to the methods previously reported [41]. The clean reads were obtained by removing adaptors and low-quality reads. To quantify small RNAs, BOWTIE software (V2.1.0) was used to map the clean reads to pig sequence from miRBase (Release 22, http://www.mirbase.org/, accessed on 10 May 2022), Genomic tRNA Database (GtRNAdb, http://gtrnadb.ucsc.edu/, accessed on 12 May 2022) [42] and tRNAscan-SE (V2.0, http://lowelab.ucsc.edu/tRNAscan-SE/, accessed on 15 May 2022) [43]. Sequence data were stored at the National Genomics Data Center (NGDC) (Accession PRJCA010563). The raw reads count are provided in Supplementary Table S1. DESeq2 (https://bioconductor.org/packages/release/bioc/html/DESeq2.html, accessed on 1 June 2022) was used to identify differential expressed miRNA (DEM) and tsRNA (DET).

### 2.5. Reverse Transcription-Quantitative PCR (RT-qPCR)

According to the manufacturer’s protocol, Mir-X™ miRNA First Strand synthesis kit (Takara, Kusatsu, Japan, Cat. #638315) was used to synthesize small RNA first-strand, and RT-qPCR was performed with TB Green Premix Ex Taq II (Takara, Cat. #RR820A) in a Bio-Rad CFX96 Real-Time PCR Detection System (Bio-Rad, Richmond, CA, USA). RT-qPCR results were normalized with U6 as an internal control and expressed as 2^−∆∆ct^. The primer sequences were as shown in Supplementary Table S2.

### 2.6. Target Gene Prediction and GO- and KEGG-Enrichment Analysis

Target genes were predicted using the online platform OmicStudio at https://www.omicstudio.cn/analysis, accessed on 2 July 2022. The target genes were subjected to Gene Ontology (GO) (http://www.geneontology.org/, accessed on 8 July 2022) and Kyoto Gene and Encyclopedia of Genomes (KEGG) (https://www.genome.jp/kegg/pathway.html, accessed on 10 July 2022) enrichment analysis. Protein–protein interaction (PPI) networks were constructed using Cytoscape (version 3.9.1, http://www.cytoscape.org/, accessed on 12 July 2022). The predicted target genes are listed in Supplementary Table S3. The results of the functional enrichment analysis are available in Supplementary Table S4.

### 2.7. Statistical Analysis

The data were analyzed using an Excel sheet in Microsoft Excel and SPSS version 26 (SPSS, IBM). Pearson’s correlation analysis was performed using the OmicStudio tools at https://www.omicstudio.cn/tool, accessed on 15 July 2022. The results are expressed as mean values ± standard error (Means ± SE). The data between the two groups were compared using a Student’s *t*-test. *p* < 0.05 was considered statistical significance and *p* < 0.01 was considered strong significance.

## 3. Results

### 3.1. Spleen and Immune Indicators of Intrauterine Growth-Restricted Pig Model

**Figure 1:**
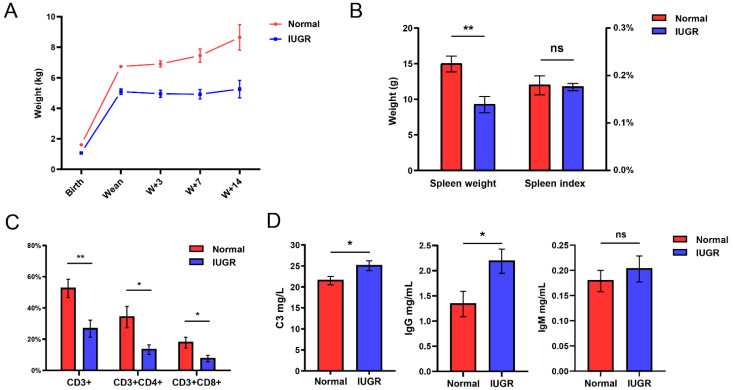
Spleen and immune indicators of intrauterine growth-restricted pigs. (**A**) The body weight of the Normal and IUGR pigs at different periods. (**B**) The spleen weight and spleen index (spleen weight as a percentage of body weight) of Normal and IUGR pigs at 14 days postweaning. (**C**) Comparison of the percentage of CD3^+^T, CD3^+^CD4^+^T and CD3^+^CD8^+^T cells in peripheral blood between Normal and IUGR pigs. (**D**) The concentration of complement component 3 (C3), IgG and IgM in plasma. The results are presented as mean ± standard error of the mean (SEM) (*n* = 6, * *p* < 0.05, ** *p* < 0.01, ns: no significant difference).

In the present study, the mean birth weight were significantly lower in IUGR piglets (1.60 ± 0.06) than normal piglets (1.07 ± 0.05) (*p* < 0.01) (Figure 1A). The IUGR pigs also had a significantly lower body weight at weaning. The body weight of IUGR pigs was not increased, but slightly declined, at 3 days and 7 days after weaning. IUGR pigs started regaining body weight until 14 days after weaning (W14) (Figure 1A). There were substantially lower for spleen weight in IUGR pigs compared with normal pigs at W14 days, but without a statistically significant difference in the spleen index (Figure 1B). Then, we analyzed the peripheral blood T-lymphocyte subsets in IUGR and normal pigs (Figure 1C). The result suggests that IUGR pigs showed significantly reduced CD3^+^ T cells, CD3^+^ CD4^+^ T cells, and CD3^+^ CD8^+^ T cells compared to normal pigs. We further detected the indicators for humoral immunity in IUGR pigs. The levels of complement C3 and immunoglobulin IgG in IUGR pigs was higher than in normal pigs at weaning (Figure 1D) (*p* < 0.05). These results show that the spleen and immune function of IUGR piglets were weak compared with normal piglets.

### 3.2. Characteristics of miRNA and tsRNA Profiling of Pig Spleen

**Figure 2:**
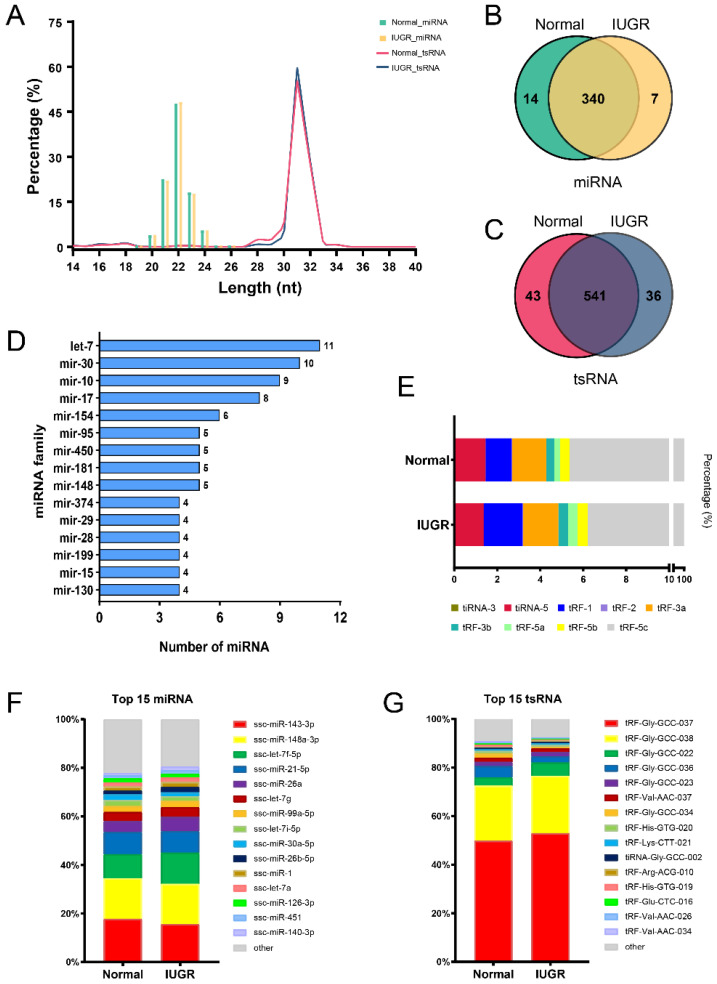
Sequence characteristics of miRNA and tsRNA profiling of pig spleen. (**A**) Length distribution of miRNA and tsRNA sequences. Venn diagram of the number of identified miRNAs (**B**) and tsRNAs (**C**). (**D**) The miRNA families of the identified miRNAs. (**E**) The types and distribution of identified tsRNAs. Composition of top 15 highly expressed miRNAs (**F**) and tsRNAs (**G**) in pig spleen.

The expression profiles of miRNA and tsRNA in pig spleen were generated by sequencing. The length of miRNAs and tsRNAs was evaluated. The length of these miRNAs was in the range of 19~26 nt and the richest was 22 nt afterwards 21 nt and 23 nt. The length range of the tsRNAs in pig spleen was wider than miRNAs and 14~40 nt (Figure 2A). A total of 361 miRNAs and 620 tsRNAs were identified in pig spleen, of which overlapping miRNAs and tsRNAs in IUGR and normal pigs account for 94.18% (Figure 2B) and 87.25% (Figure 2C) of the totals, respectively. Further analyses identified that the miRNA families let-7, miR-30, miR-10, miR-17 and miR-154 were the ones with most numerous members (Figure 2D). By analyzing the tsRNA classes, we found that tRF-5c was the most abundant class and accounted for over 90% (Figure 2E). The four most abundant miRNAs in pig spleen were ssc-miR-143-3p, ssc-miR-148a-3p, ssc-let-7f-5p and ssc-miR-21-5p, which represented more than 55% of the total sequences (Figure 2F). tRNA-Gly-GCC-derived small RNAs were most abundant in the pig spleen, of which tRF-Gly-GCC-037 and tRF-Gly-GCC-038 accounted for over 70% (Figure 2G).

### 3.3. Differentially Expressed miRNAs and tsRNAs between IUGR and Normal Pig Spleen

**Figure 3:**
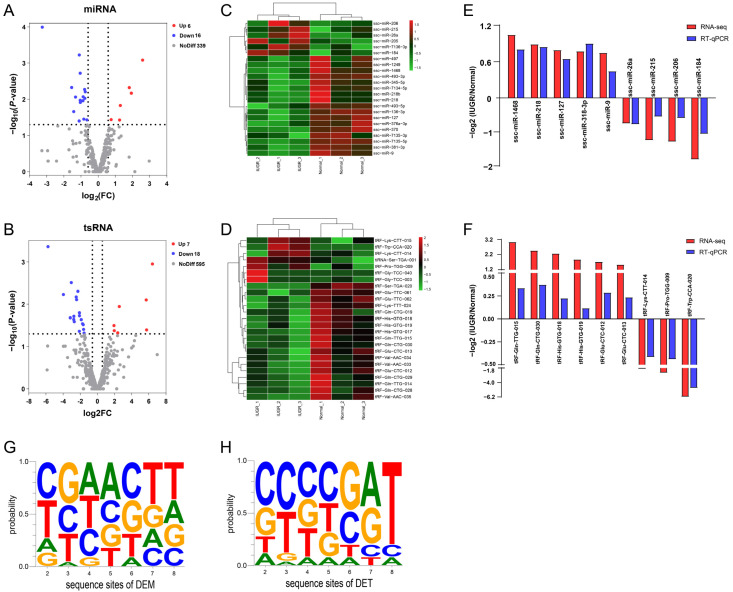
Identification of differentially expressed miRNAs (DEM) and tsRNAs (DET). Volcano plot of miRNA (**A**) and tsRNA (**B**) expression profile. Red circles represent upregulated miRNAs and tsRNAs in IUGR relative to Normal pigs. Blue circles represent upregulated miRNAs and tsRNAs in IUGR relative to Normal pigs. Gray circles represent not significantly different. Heatmap and clustering of DEMs (**C**) and DETs (**D**). RT-qPCR validation analysis of DEMs (**E**) and DETs (**F**). The basis characteristics of seed sequences for DEM (**G**) and DET (**H**).

**Figure 4:**
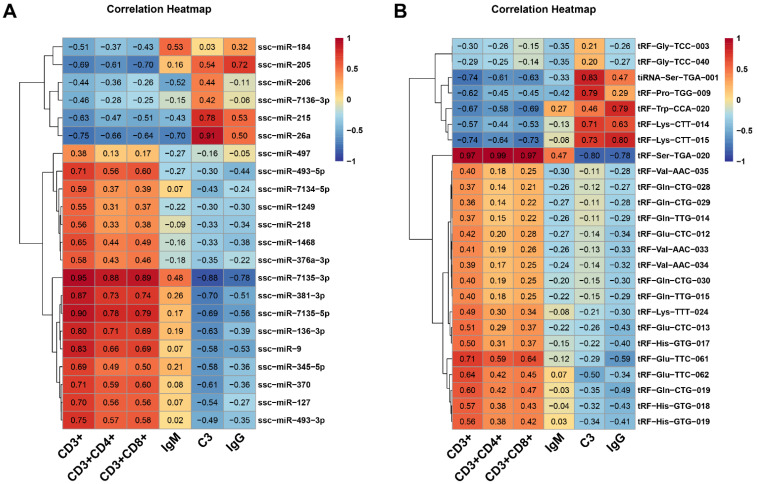
Correlation heatmap between the levels of immune-related indexes and DME (**A**) and DMT (**B**).

The miRNA and tsRNA in pig spleen associated intrauterine growth-restricted differential expression patterns were identified using DESeq2. Selection criteria were defined as Fold change ≥ 1.5 and *p*-value < 0.05 to identify DEMs and DETs. We identified 22 differentially expressed miRNAs between Normal and IUGR pig spleens. Among these 22 miRNAs, 6 and 16 were up-regulated and down-regulated in IUGR pigs, respectively (Figure 3A). In addition, a total of 25 differentially expressed tsRNAs were screened out, of which 7 were up-regulated and 18 were down-regulated (Figure 3B). Figure 3C,D present the heat map diagrams for the DEMs and DETs and hierarchical clustering demonstrated that the IUGR pigs samples clustered together. RT-qPCR was performed on nine randomly selected DEMs and DETs to verify the RNA-seq data. Both methods showed a similar trend in miRNA and tsRNA expression changes (Figure 3E,F). Moreover, we analyzed nucleotide bias at seed sequences position of DEMs and DETs (Figure 3G,H). The results reveal that the base of seed sequence varied between DEMs and DETs. Table 1 and Table 2 list the differentially expressed miRNAs and tsRNAs meeting the screen criteria. Among them, ssc-miR-26a and tRF-His-GTG-019 showed the highest expression abundance. We also analyzed the correlations between the levels of immune-related indexes and DME and DMT (Figure 4). We found weak correlations between IgM and most DEM/DET. ssc-miR-7135-3p and tRF-Ser-TGA-020 showed a strong correlation with CD3^+^, CD3^+^CD4^+^, CD3^+^CD8^+^, C3 and IgG (>0.75 in absolute value terms, Figure 4).

### 3.4. Prediction of Target Genes and Functional Enrichment Analysis of Differentially Expressed miRNAs and tsRNAs

**Figure 5:**
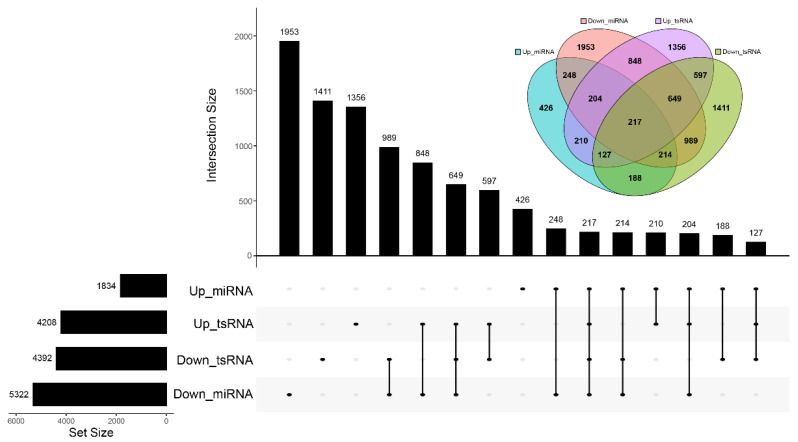
The number of predicted target genes for differentially expressed miRNAs and tsRNAs. On the left, the number of independent target genes is indicated. The middle part represents the intersection of the four sets of data. The Venn diagram summarizes the number of shared predicted genes of the four groups.

Previous several studies suggests that tsRNAs could exert similar biological mechanism as miRNA and regulate gene expression. We predicted the target genes of top 14 DEMs and top 12 DETs. Down-regulated miRNAs had the most predicted 5322 target genes and 2069 common target genes with down-regulated tsRNAs. Up-regulated miRNAs had the least predicted 1834 target genes and 210 common target genes with up-regulated tsRNAs (Figure 5).

**Figure 6:**
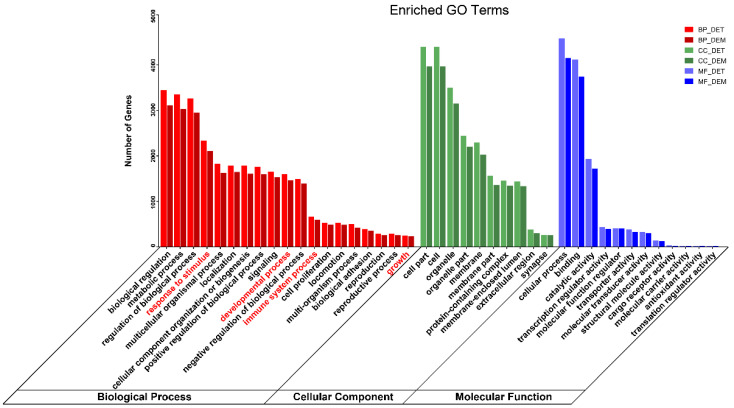
Gene ontology (GO)-enrichment analysis for differentially expressed miRNAs and tsRNAs. The red font represents important biological processes associated with IUGR.

**Figure 7:**
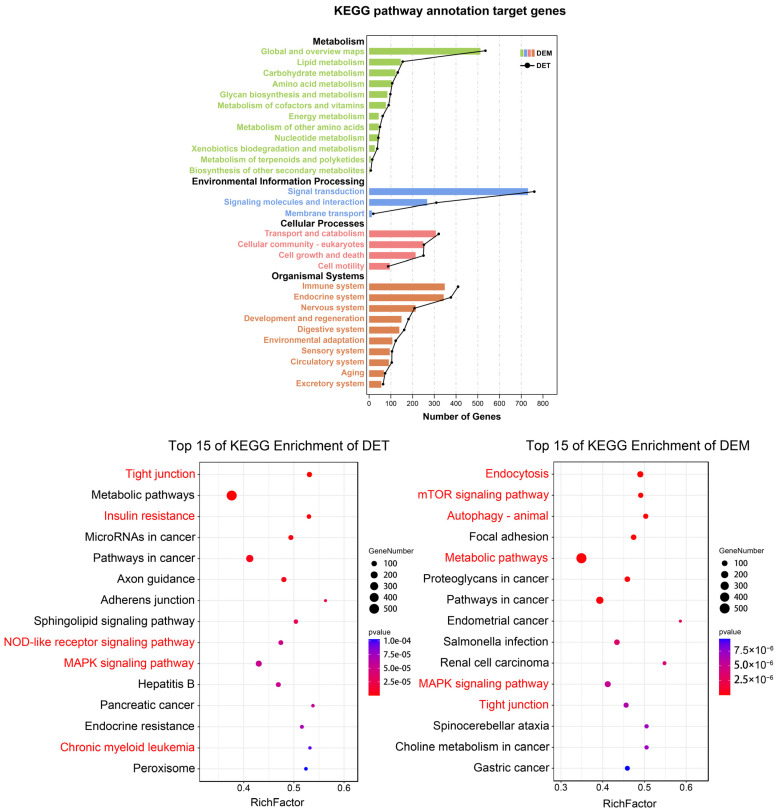
Kyoto Encyclopedia of Genes and Genomes (KEGG)-enrichment analysis for differentially expressed miRNAs and tsRNAs.

We then performed gene ontology (GO) and Kyoto Encyclopedia of Genes and Genomes (KEGG) terms analysis on these predicted target genes. GO enrichment analysis suggested that these DEMs and DMTs were involved in biological processes, including response to stimulus, developmental process, immune system process and growth. In cellular component terms, the cell part and organelle were the most significantly enriched terms. In molecular function category, the cellular process, binding and catalytic activity were the most significantly enriched terms (Figure 6). KEGG enrichment results suggest that the DETs were primarily involved in insulin resistance, sphingolipid signaling pathway and NOD-like receptor signaling pathway. The DEMs were mainly enriched in endocytosis, mTOR signaling pathway and autophagy pathways. In addition, the two share common signaling pathways, such as tight junction, metabolic pathways and MAPK signaling pathway (Figure 7).

### 3.5. Protein–Protein Interaction (PPI) Network Construction of Target Genes

**Figure 8:**
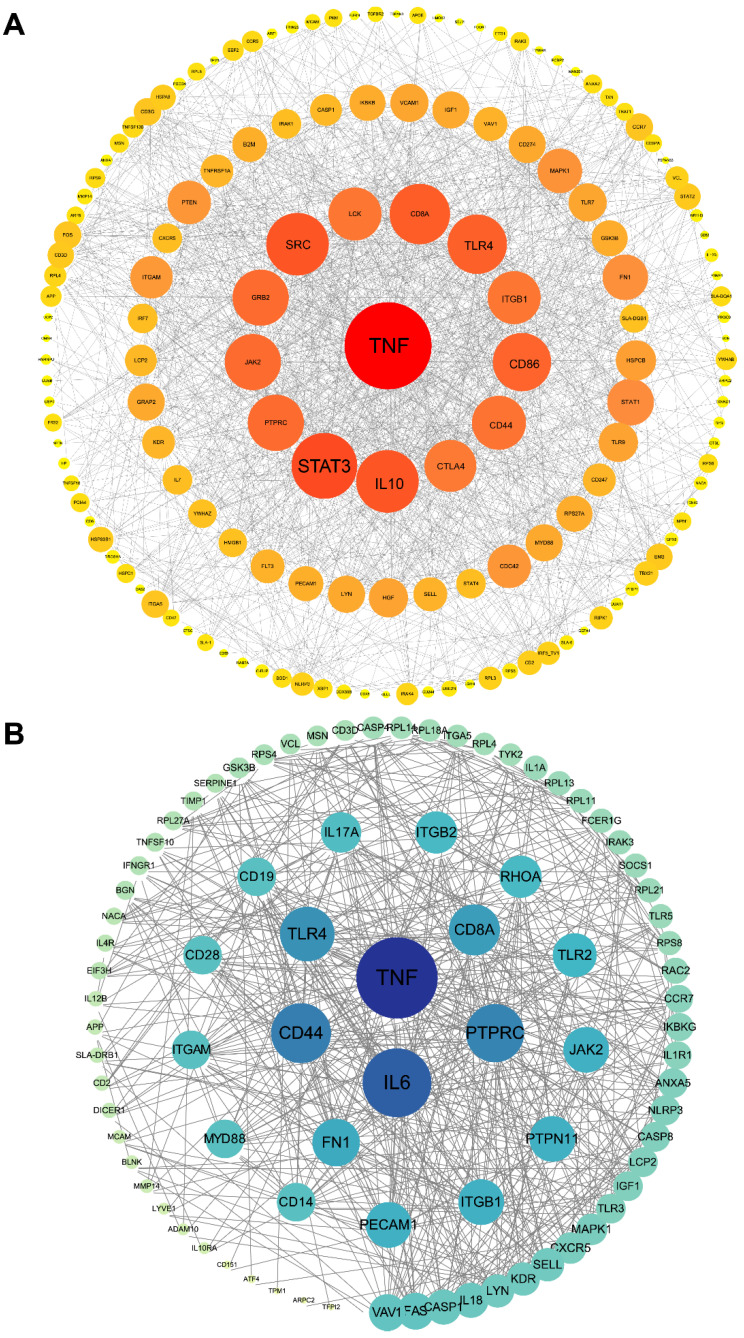
Protein–protein interaction network of target genes. (**A**) shows the interaction network of DEM target genes. (**B**) shows the interaction network of DET target genes. Each circle node represents a gene. The circle size and color scales represent the node degree of the target genes.

Further, we constructed a PPI network for target genes using STRING database. Figure 8 illustrated the interaction of target genes of DEMs and DETs, respectively. After single nodes have been ruled out, the network of DEM targets was composed of 152 nodes and 1309 edges and DET targets interaction network was 85 nodes and 555 edges. The tow PPI network showed several shared hub genes, including TNF (tumor necrosis factor), CD8A (CD8a molecule), TLR4 (Toll-like receptor 4), JAK2 (Janus kinase 2), MAPK1 (mitogen-activated protein kinase 1), IGF1 (insulin-like growth factor 1), MYD88 (MYD88 innate immune signal transduction adaptor) and ITGB1 (integrin subunit beta 1).

### 3.6. Pathway Regulation Network Related to the Immune System of IUGR Pigs

**Figure 9:**
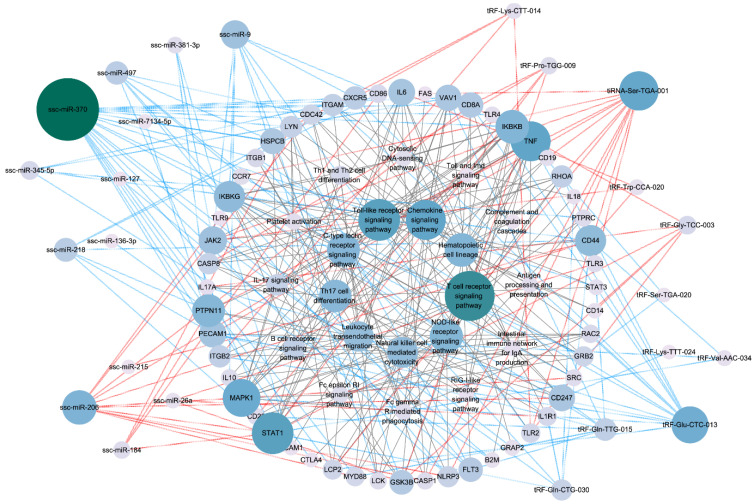
The interaction network of miRNA/tsRNA-target-gene pathway. Red lines represent up-regulated miRNAs/tsRNAs and blue lines represent down-regulated miRNAs/tsRNAs. The circle size and color scales represent the node degree.

To further investigate the key regulator of immune system pathways of IUGR pigs, we constructed an interaction network among miRNA/tsRNA, target genes and immune-related pathways (Figure 9). Most of target genes were regulated by ssc-miR-370, ssc-miR-208, tiRNA-Ser-TGA-001 and tRF-Glu-CTC-013. ssc-miR-370 have the most abundant target genes up to 21 accounting for 40.38% of the entire network. This network demonstrated several core genes of immune-related pathways, including TNF, MAPK1, STAT1 (signal transducer and activator of transcription 1) and IKBKB (inhibitor of nuclear factor kappa B kinase submit beta). T-cell receptor signaling pathway and Toll-like receptor signaling pathway were the most enriched pathway of the network.

## 4. Discussion

With respect to non-coding RNA research, tsRNA have recently emerged as a hot topic. tsRNAs can function as a vital regulator in a broad range of biological processes, such as cell proliferation and apoptosis, regulation of translation and immune responses [36,44]. More recently, the role of tsRNAs in the development of several diseases were reported [32,45]. tsRNA is valuable for disease diagnosis and treatment as potential biomarkers. In comparison to other non-coding RNAs, such as miRNA, the current tsRNA research remains in its infancy and have focused on the field of cancer. IUGR has become a global public health problem, especially in developing countries [46]. Slow growth and development and impairment of organ function are typical features for IUGR newborns, and accompanied by the increasing susceptibility towards diseases in adulthood [47,48]. Approximately 10% of human newborns have IUGR, which is close to the incidence of IUGR pigs (approximately 15%) [5]. Moreover, there are high similarities in anatomy, physiology and immunology between pigs and humans [49], and easy availability for IUGR pigs. Hence, pigs are an ideal biomedical model for studying human IUGR [50]. In this work, pigs were used as a model to investigate the characteristic and function of tsRNAs and miRNAs associated with immunocompromise caused by IUGR. This will help to broaden our understanding of the molecular mechanism of IUGR formation.

IUGR are commonly associated with decreased organ size and impaired physical function for fetus or newborns. Our results show that IUGR piglets present obviously slow growth before weaning, compared to normal-birth-weight piglets (Figure 1A). This shows that the IUGR model we used is successful and can be applied in subsequent studies. The analysis of the peripheral blood T-lymphocyte subsets showed that the proportion of CD3^+^, CD3^+^CD4^+^ and CD3^+^CD8^+^ T cells decrease in IUGR pigs. Cytokine analysis showed that pigs with IUGR had increased plasma levels of C3 and IgG at weaning. This may be because IUGR pigs with impaired immune systems are in a prolonged immune system activation state after birth to adapt to environmental changes. A previous study on reported that IUGR children had a lower percentage of CD3 T cells and a higher C3 level in plasma [51], which were in accordance with our results. The above results indicated that the function of cellular immunity and humoral immunity were disordered in our IUGR pig model.

We further evaluated tsRNA and miRNA expression profile of pig spleen by high-throughput sequencing technology. We identified 620 tsRNAs and 361 miRNAs in pig spleen. The mature or precursor tRNAs are cleaved by the specific nuclease to generate tsRNAs [36]. One tRNA commonly can be cleaved at different sites to generate multiple tsRNAs [52]. So, the number of tsRNA was more abundant. In addition, ssc-miR-143-3p, ssc-miR-148a-3p, ssc-let-7f-5p and ssc-miR-21-5p were the top four most abundant expressed miRNAs, accounting for more than 50%. Zheng et al. reported miR-148a-3p can impair CD8^+^ T-cell-mediated immune attack [53]. miRNA-148a-3p also regulates immunosuppression by targeting PD-L1 [54]. Gao et al. reported that miR-21-5p was involved in regulating the immune response triggered by CPB2 toxin [55]. miR-21-5p also may be involved in fetoplacental growth [56]. tRF-Gly-GCC-037 and tRF-Gly-GCC-037 showed the highest expression abundance in the pig spleen, accounting for more than 70%, which both were from tRNA-Gly (GCC). Their higher expression may play a significant role in IUGR and immunoregulatory. tsRNAs can be categorized into two classes [57]. The mature tRNA was cleaved at the anticodon site by angiogenin (a ribonuclease) to generate fragments termed as tiRNAs [57]. Other category of tsRNAs is referred to collectively as tRNA-derived small fragments (tRFs). According to the different digesting positions and fragment lengths, they can be divided into tRF-1, tRF-2, tRF-3 and tRF-5. tRF-3 and tRF-5 can be further subdivided into tRF-3a, tRF-3b, tRF-5a, tRF-5b and tRF-5c [58]. In this study, we find that tRF-5c were the most abundant categories in pig spleen. Previous studies showed that the class of tsRNA in stomach [59], lung [60] and plasma [61] were mainly tRF-5c. However, other results were also reported tiRNAs were primarily observed in ovary [62] and thyroid [63]. These suggest that tsRNAs may have tissue-specific expression.

We then used DEseq2 to identify DEMs and DETs between IUGR and normal pigs. The RNA-seq data was validated by RT-qPCR. It is already clear that tsRNAs are small fragments derived from cleaved tRNAs. tsRNAs have been proven not to be products of random degradation, but is a type of non-coding RNA that has regulatory functions. Growing evidence suggests that tsRNA is present in almost all cell types [36]. Recent evidence has suggested that Argonaute (AGO) family proteins can recruit tsRNA to regulate gene expression at the post-transcriptional level [64]. Green et al. [65] reported that tRF-3003a could bind to the JAK3 3′-UTR to inhibit JAK3 expression in chondrocytes. Zhong et al. reported that Gly-tRF inhibit the translation of Sirt1 mRNAs through binding to complementary sequences in 3′ UTR to promote hepatosteatosis. In addition, tRNAGlu-derived fragment was proved to be able to suppress breast cancer progression by targeting nucleolin [66]. Given such evidence, we performed target gene prediction and functional enrichment analysis on DETs by a similar approach with miRNA. GO analysis showed that DEMs and DETs were mainly enriched in response to stimulus, developmental process, immune system process and growth terms. This suggests the target genes of DEMs and DETs are associated with the growth and immune system of IUGR pigs. Further KEGG pathway analyses showed that DETs were mainly involved in insulin resistance, the NOD-like receptor signaling pathway and sphingolipid signaling pathway. The DEMs were mainly enriched in endocytosis, the mTOR signaling pathway and autophagy. Notably, we observed the common enriched pathway between DETs and DEMs, such as tight junction, MAPK signaling pathway and metabolic pathway. 

It has been reported that the NOD-like receptor signaling pathway [67], tight junction [17] and MAPK signaling pathway [68] are related to the occurrence of IUGR. 

The mTOR signaling pathway [69], endocytosis and autophagy [70] are related to immune function in mammals. The functional enrichment analysis of these two small RNA produced similar results. This indicates that DETs and DEMs may play an important role in the formation of IUGR.

A PPI network analysis was also performed on these DEMs and DETs. We noted that there were some common core genes, such as TNF, TLR4, CD44, etc. A study reported TNF was involved in nuclear factor kappa B (NF-κB)-mediated innate immune response to inflammatory stimuli in IUGR mice [71]. Chan et al. reported that the mutation of TLR4 led to IUGR and abortion, which was associated with maternal immune disorders [72]. All these results point to DEMs and DETs involved the regulation of the immune system of IUGR pigs. Based on the above results, we constructed a miRNA/tsRNA-hub genes pathway regulatory network associated with the immune system. ssc-miR-370, ssc-miR-206, tiRNA-Ser-TGA-001 and tRF-Val-AAC-034 were important regulators of interaction networks. As reported, microRNA-206 can drive the M1 polarization of Kupffer cells and promoted the recruitment of CD8^+^ T cells in mice [73]. In addition, miR-206 in T cells was also potential biomarkers for Th17-type immune reactions in mouse [74]. These results prove that miR-206 is able to regulate the immune system. TNF, MAPK1 and STAT1 were important bridge nodes of networks. T-cell receptor signaling pathway was the most important one that DEMs and DETs participate in, followed by Toll-like receptor signaling pathway and chemokine signaling pathway. In terms of tsRNA, studies on tsRNAs are still lacking, and studies on associating IUGR are even rarer. These differentially expressed tsRNAs possess strong potential for research. The regulation pathways revealed by this network can serve as research targets in future verifications in animal models.

## 5. Conclusions

In conclusion, the present study provided the first catalog of the expression profiling of tsRNAs and miRNAs in spleen in the IUGR-affected pigs in this disease model. The majority of the tsRNA identified belonged to tRF-5c. We identified 25 and 22 differentially expressed tsRNAs and miRNAs, respectively. Functional enrichment analysis revealed the DETs and DEMs might be involved in the compromised immune parameters associated with IUGR. Our results provide new evidence that miRNAs/tsRNAs are involved in IUGR pig spleen immunity and provide a basis for the future research on IUGR in other mammals.

## Figures and Tables

**Table 1:** List of miRNA populations differentially expressed in the spleen between Normal and IUGR pigs.

Type	miRNA-ID	Normal-CMP	IUGR-CMP	log2FC	*p*-Value
Up-regulated	ssc-miR-205	0.1883	1.0705	2.5905	0.000825628
	ssc-miR-7136-3p	0.5789	1.7741	1.9331	0.006799598
	ssc-miR-184	6.299	19.002	1.8141	0.004753366
	ssc-miR-206	17.486	32.298	1.2932	0.014926584
	ssc-miR-215	15.401	28.950	1.2511	0.037612553
	ssc-miR-26a	44735	58503	0.7552	0.036464342
Down-regulated	ssc-miR-7135-5p	1.7123	0.1257	−3.2535	0.000101718
	ssc-miR-1249	3.3335	0.8842	−1.5453	0.004733207
	ssc-miR-493-5p	4.4964	1.2656	−1.4257	0.021899034
	ssc-miR-376a-3p	4.4698	1.3902	−1.3168	0.008688021
	ssc-miR-7135-3p	4.0429	1.5163	−1.1024	0.039710544
	ssc-miR-370	39.167	14.533	−1.0981	0.000597613
	ssc-miR-345-5p	16.812	6.615	−1.0461	0.010609881
	ssc-miR-1468	282.04	105.31	−1.0445	0.00191462
	ssc-miR-493-3p	4.7686	1.8293	−1.0411	0.012239605
	ssc-miR-218	1788.05	750.36	−0.8888	0.01102262
	ssc-miR-136-3p	25.72	11.29	−0.8684	0.008511165
	ssc-miR-497	40.94	17.7	−0.8377	0.035318563
	ssc-miR-127	319.23	147.31	−0.7924	0.005334554
	ssc-miR-381-3p	6486.27	3045.71	−0.7757	0.009420996
	ssc-miR-9	640.91	300.39	−0.7493	0.006231918
	ssc-miR-7134-5p	107.070	54.293	−0.6539	0.037300766

Note: CPM, counts per million-mapped reads. log2FC, log2 (Fold-Change). The positive value of log2FC was up-regulated in IUGR relative to Normal pigs.

**Table 2:** List of tsRNA populations differentially expressed in the spleen between Normal and IUGR pigs.

Type	tsRNA-ID	Normal-CMP	IUGR-CMP	log2FC	*p*-Value
Up-regulated	tRF-Trp-CCA-020	0	48.6523	6.4292	0.001116986
	tRF-Gly-TCC-040	0	18.0276	5.7421	0.040442328
	tRF-Gly-TCC-003	10.90	408.74	5.7054	0.007916416
	tRF-Pro-TGG-009	6.263	40.205	2.5519	0.011326413
	tRF-Lys-CTT-015	2.098	21.47	2.3787	0.047462016
	tiRNA-Ser-TGA-001	6.282	26.298	1.9759	0.042137556
	tRF-Lys-CTT-014	14.178	72.018	1.9456	0.031865939
Down-regulated	tRF-Ser-TGA-020	23.454	0	−5.7568	0.000436346
	tRF-Gln-CTG-028	70.232	2.902	−3.9634	0.00590216
	tRF-Gln-CTG-029	233.094	18.174	−3.1648	0.020822792
	tRF-Gln-TTG-015	300.411	29.932	−3.0391	0.003070212
	tRF-Gln-TTG-014	232.12	25.72	−2.7517	0.022836512
	tRF-Gln-CTG-019	98.760	10.323	−2.7407	0.019339968
	tRF-Glu-TTC-062	21.424	3.230	−2.6897	0.025766303
	tRF-Gln-CTG-030	964.27	145.702	−2.4727	0.006773031
	tRF-Val-AAC-033	713	106	−2.425	0.007834966
	tRF-His-GTG-018	1198	223	−2.2756	0.004945062
	tRF-Glu-TTC-061	33.508	8.631	−2.1028	0.043496743
	tRF-Val-AAC-034	4270	789	−2.0977	0.018732543
	tRF-Val-AAC-035	85.789	20.367	−2.0953	0.016049707
	tRF-His-GTG-019	6449	1594	−1.8803	0.02306075
	tRF-Glu-CTC-012	2202	621	−1.7247	0.028886881
	tRF-His-GTG-017	490	144	−1.6523	0.030190001
	tRF-Lys-TTT-024	112.961	41.084	−1.6095	0.039361651
	tRF-Glu-CTC-013	3520.92	1146.79	−1.5432	0.048273186

Note: CPM, counts per million-mapped reads. log2FC, log2 (Fold-Change). The positive value of log2FC was up-regulated in IUGR relative to Normal pigs.

## Data Availability

RNA-Seq raw reads have been deposited in the National Genomics Data Center accession PRJCA010563. For the remaining data that may be relevant, the corresponding authors can be contacted.

## Supplementary Materials

The following supporting information can be downloaded at: https://www.mdpi.com/article/10.3390/ani12162102/s1, Table S1: Read counts; Table S2: Primer sequence; Table S3: Predicted target genes; Table S4: Results of GO and KEGG enrichment analyses.
